# Database Citation in Full Text Biomedical Articles

**DOI:** 10.1371/journal.pone.0063184

**Published:** 2013-05-29

**Authors:** Şenay Kafkas, Jee-Hyub Kim, Johanna R. McEntyre

**Affiliations:** European Molecular Biology Laboratory – European Bioinformatics Institute Wellcome Trust Genome Campus, Cambridge, United Kingdom; Université de Montréal, Canada

## Abstract

Molecular biology and literature databases represent essential infrastructure for life science research. Effective integration of these data resources requires that there are structured cross-references at the level of individual articles and biological records. Here, we describe the current patterns of how database entries are cited in research articles, based on analysis of the full text Open Access articles available from Europe PMC. Focusing on citation of entries in the European Nucleotide Archive (ENA), UniProt and Protein Data Bank, Europe (PDBe), we demonstrate that text mining doubles the number of structured annotations of database record citations supplied in journal articles by publishers. Many thousands of new literature-database relationships are found by text mining, since these relationships are also not present in the set of articles cited by database records. We recommend that structured annotation of database records in articles is extended to other databases, such as ArrayExpress and Pfam, entries from which are also cited widely in the literature. The very high precision and high-throughput of this text-mining pipeline makes this activity possible both accurately and at low cost, which will allow the development of new integrated data services.

## Introduction

Linking the scientific literature to databases is a goal pursued by many sectors of the scientific community, driven by the need to enable scientists to navigate and analyse research information in a timely and comprehensive manner.

In the field of biological sciences, there is a long tradition of partnership between journals and public databases, ensuring that data are archived and available for reuse in the long term. This began with an agreement between the EMBL Data Library and the journal Nucleic Acids Research (NAR) in 1988 [1]. In this article, Kahn and Hazledine outlined the new NAR policy, which was that any article that discussed or contained sequence data was required to show evidence that the sequence data has been deposited in the EMBL Data Library, i.e. the data had to be cited using an accession number. This approach to data management was subsequently adopted widely by biological science journals and has since been applied to other types of data including protein structures and gene expression experiments, becoming standard practice in many areas of research.

Data cited by, embedded in, or otherwise associated with research articles is a topic of widening interest for a number of reasons. As research becomes more data intensive, and, in the interests of transparency and access to the results of publicly funded research [2], funders are increasingly mandating that data sets as well as full text articles should be open and free to reuse (http://www.rcuk.ac.uk/research/Pages/outputs.aspx). Furthermore, measures of research impact that complement established methods (such as those based on article publication and citation articles), are now being explored. The publication of data and evidence of its reuse (i.e. citation) by the community could be a valid additional measure of the impact of a piece of research. Indeed, Thomson ISI, home of the Impact Factor, recently announced the launch of a new product, the Data Citation Index (http://thomsonreuters.com/content/press_room/science/730914).

In this context, it would be informative and useful to analyse current data citation practices in the life sciences literature. In order to reuse information about data citation effectively (i.e. compute on it) data citations in full text have to be structured in the archival record of the article, just as article reference lists are currently. This has become possible since XML technologies were introduced and widely adopted in journal publishing.

If data accession numbers are not available in structured form, text-mining is a useful approach to extract this information instead. For example, Whatizit [3] (http://www.ebi.ac.uk/webservices/whatizit/info.jsf) is a text-mining service that analyses publications and links them to external biological databases based on named entity recognition modules for selected semantic types (e.g. gene/protein names, diseases, organisms). Whatizit is integrated into the Europe PMC infrastructure (http://europepmc.org/) and is used to tag these entities in full text articles on a daily basis [4]. Included in this system is a module for recognising and resolving a variety of database accession numbers. This information is not only useful for tracking data citation, but also as a source of further enrichment of the source database content.

A few studies have focused on automatically finding data citation in articles. For example, Névéol and colleagues used machine learning techniques to identify sentences containing data deposition statements [5], [6]. The BioLit portal provides clickable links from full-text articles to PDB and Gene Ontology (http://www.geneontology.org) based on accession numbers identified in the text [7]. Such numbers provide an explicit way to link publications with biological databases. A slightly different approach to finding new literature-data links was used by Haeussler *et al*., when they mined DNA sequences from full text and used these to link to genomic sequences in Ensembl [8].

**Figure 1:**
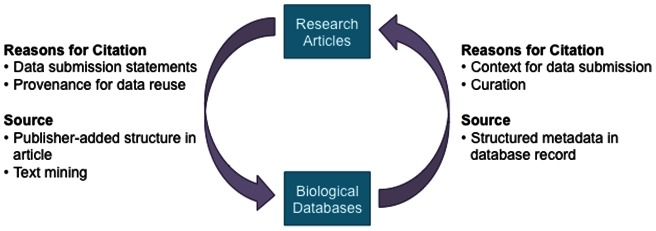
The reciprocal citation relationships between articles and database records.

Further relationships between data and articles can be gleaned via database submissions. On submission of data to a database (e.g. European Nucleotide Archive (ENA)), it is strongly encouraged to provide a reference to a published or accepted article that supports the database submission. In secondary databases such as Universal Protein Knowledgebase (UniProt), database curators annotate database records with biological information and cite the source articles as references. When there is no full text available for text mining accession numbers, these database-derived cross-references can be used as an alternative source of linking articles to databases (Figure 1).

In this study, we explore a number of questions regarding data citation practice and management in articles, and compare these findings with data-literature crosslinks from several databases. We explore the scope of structured data citation mark-up by publishers, and to what extent text-mining adds value to this activity.

To the best of our knowledge, this is the first comprehensive study on accession number citation analysis in the Open Access subset of Europe PubMed Central (OA-ePMC) full text articles. The annotated set of OA-ePMC articles used for this analysis is publically available at http://europepmc.org/ftp/oa/AccNoAnalysisData/.

We focus on article-to-database and database-to-article citations between OA-ePMC articles and three major databases: the European Nucleotide Archive (ENA, http://www.ebi.ac.uk/ena/), UniProt (http://www.uniprot.org/) and Protein Data Bank (Europe) (PDBe, http://www.ebi.ac.uk/pdbe/). The three key citation sets were as follows:

1. Accession numbers available in articles as publisher-supplied, structured content;
2. Accession numbers identified in articles by text mining;
3. References to articles from the ENA, UniProt and PDBe records.

## Materials and Methods

### Literature and Biological Databases Used

The full-text articles used in this study were gathered from the Open Access subset of Europe PubMed Central (OA-ePMC). Europe PubMed Central (Europe PMC) is one of the international nodes based on PubMed Central (PMC), the digital archive of biomedical and life sciences journal literature based at the National Library of Medicine (NLM), in the USA. Europe PMC provides a variety of ways to access 26 million abstracts (including all of PubMed) and 2.5 million full text articles. The full text component is available in a number of formats: XML, OCR text extracted from PDF images, and PDF-text files. Only a portion of this corpus is open access i.e., available for reuse such as for text mining. These articles are available at http://europepmc.org/ftp/oa.

**Figure 2:**
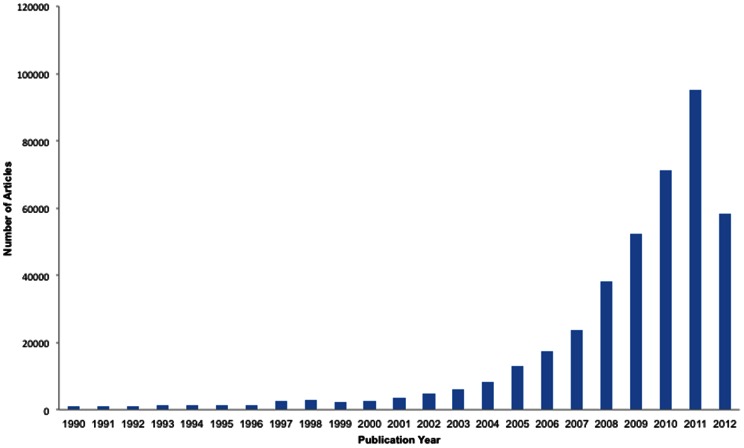
Distribution of number of articles according to years in the OA-ePMC set. This figure shows the distribution of articles by publication year in the OA-ePMC set. Note the apparent decrease in OA articles available in 2012 is due to an incomplete year (dataset was frozen for this study in June 2012).

For the purposes of this study, we limited the corpus to open access articles available as full text XML, since only these could contain structured (“tagged”) database accession number. As of June 2012, this dataset contained a total of 486,472 articles. We also filtered out articles published as OA XML before 1990, because in this historical set, accession number citations are rare and more likely to be false positives with respect to text-mining PDBe, ENA and UniProt accession numbers. This resulted in a set of 410,364 articles, which has been used throughout this study and is available in full, with all text-mined accession number annotations, from here: http://europepmc.org/ftp/oa/AccNoAnalysisData/. Figure 2 shows the distribution, by publication date, of articles in this sample set. As can be seen from the figure, the bulk of OA articles have been published in the past ∼10 years.

How representative is this set of all of the content of Europe PMC? OA articles are a growing portion of the intake of content into Europe PMC year-on-year, with the past year or two seeing 40–50% of articles being OA (see: http://blog.europepmc.org/2012/05/increasing-proportion-of-ukpmc-articles.html). So, as a subset of recent Europe PubMed content, this 410 K set of articles is likely a reasonable proxy for the content of the database as a whole. A more difficult question to address is “How representative is this set of all of the life science publishing output?”. This is particularly true for the accession number mining outlined in this article, as citing accession numbers in abstracts is extremely rare therefore abstracts cannot be used as a proxy for the full text for this purpose. This also illustrates the necessity of OA for analysing data citation practice and possible future development of metrics for data citation.

Europe PMC was used as the source of links to databases from PubMed abstracts. Europe PMC contains database links by gathering ID pairs (e.g. UniProt ID – PMID) generated by submitters to and curators of biological databases, and then making the reverse link available.

The comparative part of this study focuses on three major databases: UniProt, ENA and PDB, Europe. These are the databases that publishers primarily tag accession numbers for, as described in the JATS DTD (http://dtd.nlm.nih.gov/publishing/tag-library/3.0/index.html). UniProt is a protein database consisting of both manually and automatically annotated protein records; ENA is a partner in the International Nucleotide Sequence Databases (INSD) Collaboration (http://www.insdc.org); PDB, Europe is an archive of macromolecular structural data and a partner in the Worldwide Protein Data Bank collaboration (http://www.wwpdb.org).

### Publisher-Annotated Accession Numbers

The submission guidelines for many journals list the databases that authors should cite, and how they should be cited. For example, *BMC Genomics* states that nucleic acid sequences, protein sequences, and the atomic coordinates of macromolecular structures should be deposited in the appropriate database, and that the accession number should be cited in the published article. The accession numbers should be provided in square brackets with their corresponding database name (e.g. [EMBL:AB026295, EMBL:AC137000, DDBJ:AE000812, GenBank:U49845, PDB:1BFM, Swiss-Prot:Q96KQ7, PIR:S66116]). However, not all journals have such a clear guideline on how to describe data submissions.

The NLM JATS DTD contains an element for describing database cross links (http://dtd.nlm.nih.gov/publishing/tag-library/3.0/index.html). The following examples demonstrate how this element is used to annotate accession numbers from the three databases that are the focus of this article.

〈ext-link ext-link-type = “pdb” xlink:href = “1EWK”〉1EWK〈/ext-link〉

〈ext-link ext-link-type = “gen” xlink:href = “AY762362”〉AY762362〈/ext-link〉

〈ext-link ext-link-type = “spr” xlink:href = “Q08943”〉Q08943〈/ext-link〉

All instances of these tags were extracted from the OA-ePMC full text articles to reveal the extent to which publishers annotate articles with explicit database accession numbers.

### Identification of Accession Numbers by Whatizit

Accession number tagging is a relatively new feature of the Whatizit annotation tool. Accession numbers are identified based upon a combination of rule-based knowledge of possible accession numbers and an empirically determined set of contextual cues. The current version of Whatizit has recently been extended to annotate Ensembl (http://www.ensembl.org/), Pfam (http://pfam.sanger.ac.uk/) [9], ArrayExpress (http://www.ebi.ac.uk/arrayexpress/) [10] and InterPro (http://www.ebi.ac.uk/interpro/) [11] accession numbers, in addition to those from ENA, UniProt, and PDB. Pfam, ArrayExpress and InterPro accession numbers are not annotated by publishers; therefore, while we report on the results of those pipelines, they are excluded from the comparative analyses carried out in this study.

Whatizit Accession Number Annotation (ANA) is completed in three steps:

1. Analysing the context of an article
2. Identifying accession numbers in each sentence and
3. Validation of the mined accession numbers.

The first step collects a list of contextual clues from the text such as the occurrence of terms GenBank (http://www.ncbi.nlm.nih.gov/genbank/) (for capturing ENA accession numbers), UniProt, PDB, accession, at the article and sentence levels. If an article-level context is found, the second step proceeds and identifies accession numbers in each sentence using regular expressions based on database accession number format guidelines (summarized in Table 1). The sentence-level context is used to decide which database type should be used for regular expressions. The final step is to validate mined accession numbers against its corresponding database. If a mined accession number is not found in the database, the number is removed to prevent false positive accession number tagging.

**Table 1:** Extraction patterns and contextual cues for databases.

Database	Patterns	Contextual Cues
ENA	1 letter +5 numerals; 2 letters +6 numerals; 3 letters +5 numerals; 4 letters +8–10 numerals; 5 letters +7 numerals	genbank, gen, ddbj, embl
UniProt	[A–N,R–Z][0–9][A–Z][A–Z, 0–9][A–Z, 0–9][0–9]; [O,P,Q][0–9][A–Z, 0–9][A–Z, 0–9][A–Z, 0–9][0–9]	swissprot, sprot, uniprot
PDB	[0–9][A–Z, 0–9][A–Z, 0–9][A–Z, 0–9]	Pdb

Patterns are separated by the “;” sign.

### Citation of articles by database records

Records in the biological databases often contain literature citations, either supplied by the authors on submission, or added by curators. These citations can be resolved to PubMed IDs, which in turn can be mapped to PMCIDs, where available. The ePMC database infrastructure maintains these relationships, using them to link back to the databases from abstracts and articles. The accession numbers from ENA, UniProt and PDB that are linked to the OA-ePMC subset were gathered for comparison with publisher-annotated and text-mined accession numbers.

## Results and Discussion

### Performance Assessment of the Accession Number Identification by Whatizit

To assess the performance of the Whatizit ANA pipeline, for each of the three databases (ENA, PDB and UniProt), we formed a Gold Standard article set by randomly selecting 50 documents that contained publisher supplied structured accession number citations to records from those databases. We carried out two types of performance assessment. First, we estimated the performance of Whatizit ANA by assuming that publisher supplied structured accession numbers in the articles are a gold standard for annotation (“automatic” assessment). Second, we manually analysed the false positive annotations which were provided from our pipeline, by reading the context of the database citation, given that structured accession numbers provided in articles might not be always complete or correct. This means that annotations made by Whatizit ANA that were not already tagged in the article would be called as false positives by the first (automatic) assessment, but on manual inspection could be reassigned as true positives. For example, the Whatizit ANA pipeline annotates ENA accession number AY311498 correctly, which were missed by the publishers in the article PMC3315791. Therefore, the results of the automatic and manual inspections shown in Table 2 are slightly different. The manual performance estimates are higher and more accurate than those obtained from the automatic evaluation as we found that not all accession numbers were correctly identified and tagged in the formed gold standard article sets. However, given that we have manually evaluated the false positive annotations only for investigating what value, in practical terms, is added by text-mining to the work already done by publishers in terms of coverage, the performance of the Whatizit ANA pipeline might be overestimated since there are accession numbers likely to be missed by the publishers and the pipeline. The annotation of accession numbers by Whatizit has been engineered for high precision (100% by this manual inspection), but perhaps at the expense of some recall, resulting in F-score values of >95% on PDB and UniProt annotations and 75.17% on ENA annotations. The manual inspection revealed that the false negative errors were due to several factors, some of which can be addressed to improve the recall:

**Table 2:** Performance assessment results of the Whatizit ANA pipeline.

Database	Evaluation	#TP	#FP	#FN	Precision	Recall	F-score
ENA	automatic	267	7	181	97.45%	59.60%	73.96%
	manual	274	0	181	100.00%	60.22%	75.17%
UniProt	automatic	569	8	39	98.61%	93.59%	96.03%
	manual	577	0	39	100.00%	93.67%	96.73%
PDB	automatic	529	30	50	94.63%	91.36%	92.97%
	manual	559	0	50	100.00%	91.79%	95.72%

True Positive (TP): A positive object that is (correctly) annotated as positive by the pipeline False Positive (FP): A negative object that is (incorrectly) annotated as positive by the pipeline False Negative (FN): A positive object that is missed by the pipeline.

Precision = TP/(TP+FP) Recall =  TP/(TP+FN) F-score = 2×Precision×Recall/(Precision+Recall).

The performance of the Whatizit ANA pipeline might be a bit overestimated since we evaluate false positive annotations only and there are accession numbers likely to be missed by the publishers and the pipeline.

1. Article footnotes are not annotated by the current version of Whatizit, as they appear in the back matter of the article and this section was deliberately omitted by the pipeline to avoid annotating reference lists. Footnotes clearly contain accession numbers. For example, the Whatizit ANA pipeline misses ENA accession number CP000155, which is publisher-tagged in a footnote of the article PMC1312362.
2. The lower performance estimate for ENA annotations is in large part due to the fact that ENA does not include the RefSeq (http://www.ncbi.nlm.nih.gov/RefSeq/) set of accession numbers. On the other hand, accession numbers from RefSeq are annotated as GenBank by publishers given that RefSeq is part of GenBank. Therefore, when candidate ENA accession numbers are identified and those candidates are RefSeq identifiers, the final resolving step to ENA would not be successful and would discard the candidate as a false positive.
3. Sometimes the Whatizit ANA pipeline only partially identifies accession numbers and fails to identify ranges. For example, in the sentence “*Hence in the present study, three templates (PDB IDs:1J4N, 1FX8 and 1RC2-B chain) were chosen ....*” (PMC1866351) our pipeline tagged 1J4N, 1FX8 and only 1RC2, not the subchain B. In another sentence, “*Sequences specifically obtained for this study have been deposited in GenBank [GenBank: EF151088–EF151123 and EF153103].*” (PMC1838906), our pipeline tagged EF151088, EF151123 and individually EF153103, as the current version does not identify ranges.
4. In some cases, there are errors regarding the actual source of the accession number and the stated source. For example, in the sentence “*Two of the sequences obtained, designated β1–1 and β1–2, had a high degree of identity to other vertebrate β1 sequences, such as human β1 [Genbank Q8WUM6] (* *Fig. 2* *).*” (PMC1538996), the accession number Q8WUM6 is annotated as a GenBank (ENA) accession number, when in fact it is a UniProt accession number, tagged as such by our pipeline, but would be ruled incorrect if we consider the publisher-source information as gold standard.

### Extension and improvement of Whatizit accession number annotation

**Figure 3:**
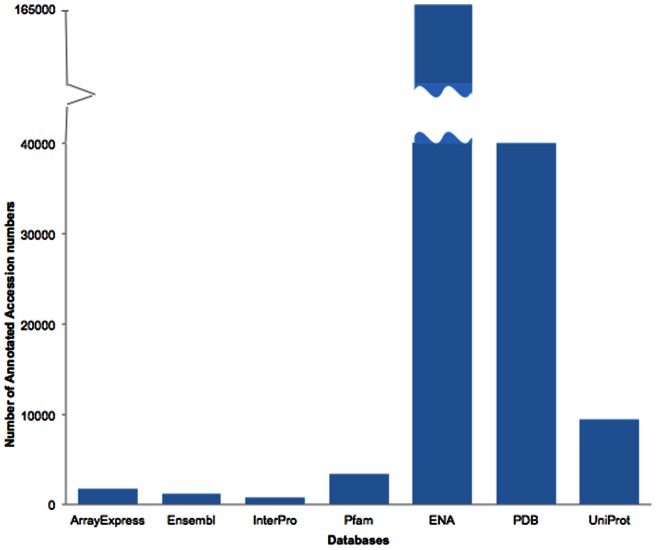
Distribution of annotated accession numbers according to database. The figure shows the distribution of accession numbers from different databases found in the OA-ePMC subset. The total numbers of annotations are as follows: ArrayExpress: 1,785; Ensembl: 1,121; InterPro:773; Pfam:3,361; ENA:160,112; PDB:39,977 and UniProt:9,430. The count for the core three databases (ENA, PDB, UniProt) represents the superset of text-mined and publisher-annotated accession numbers, while for the other databases the numbers represent text-mined statistics only, given that publishers do not annotate these accession numbers.

In addition to ENA, PDB and UniProt accession numbers, Whatizit was extended to mine ArrayExpress, Ensembl, Pfam and InterPro accession numbers to explore the value of widening the scope to reflect scientific advances over the past few years. Figure 3 shows the annotated accession numbers' distribution according to the submission databases.

These initial results suggest that, given the volume of references found, and the low cost and high precision of the text-mining method we deploy (Table 2), it is useful to extend the scope of accession number mining beyond the “core three” data sources that publishers currently mark up. While references to ENA/GenBank and PDB remain the most numerous by far, the volume of references to Ensembl, ArrayExpress and Pfam are comparable with those to UniProt.

The manual examination of the initial outputs described in this article revealed several ways in which the Whatizit ANA pipeline could be improved, such as extending the annotation methods to capture RefSeq citations, operating on footnotes, and capturing subchains and ranges of accession numbers more effectively. Some of these improvements are already underway, and the new pipelines will be deployed across all of the content of Europe PubMed Central. The results of the Whatizit ANA pipeline will be available on an ongoing basis via the Europe PubMed Central website and web services.

### Analysis of the publisher-annotated and text-mined accession numbers

As described in the methods section, the subset of 410,364 full text XML articles, were used for this analysis. The bulk of these have been published in the past 10 years.

Overall, only 2.26% (9,268/410,364) of the articles were annotated with explicit database accession numbers for ENA, UniProt and PDB by publishers. This number was improved to 5.15% (21,119/410,364) by using the text mining techniques for ENA, UniProt and PDB accession numbers described in the methods and materials section to supplement publisher tagging.

**Figure 4:**
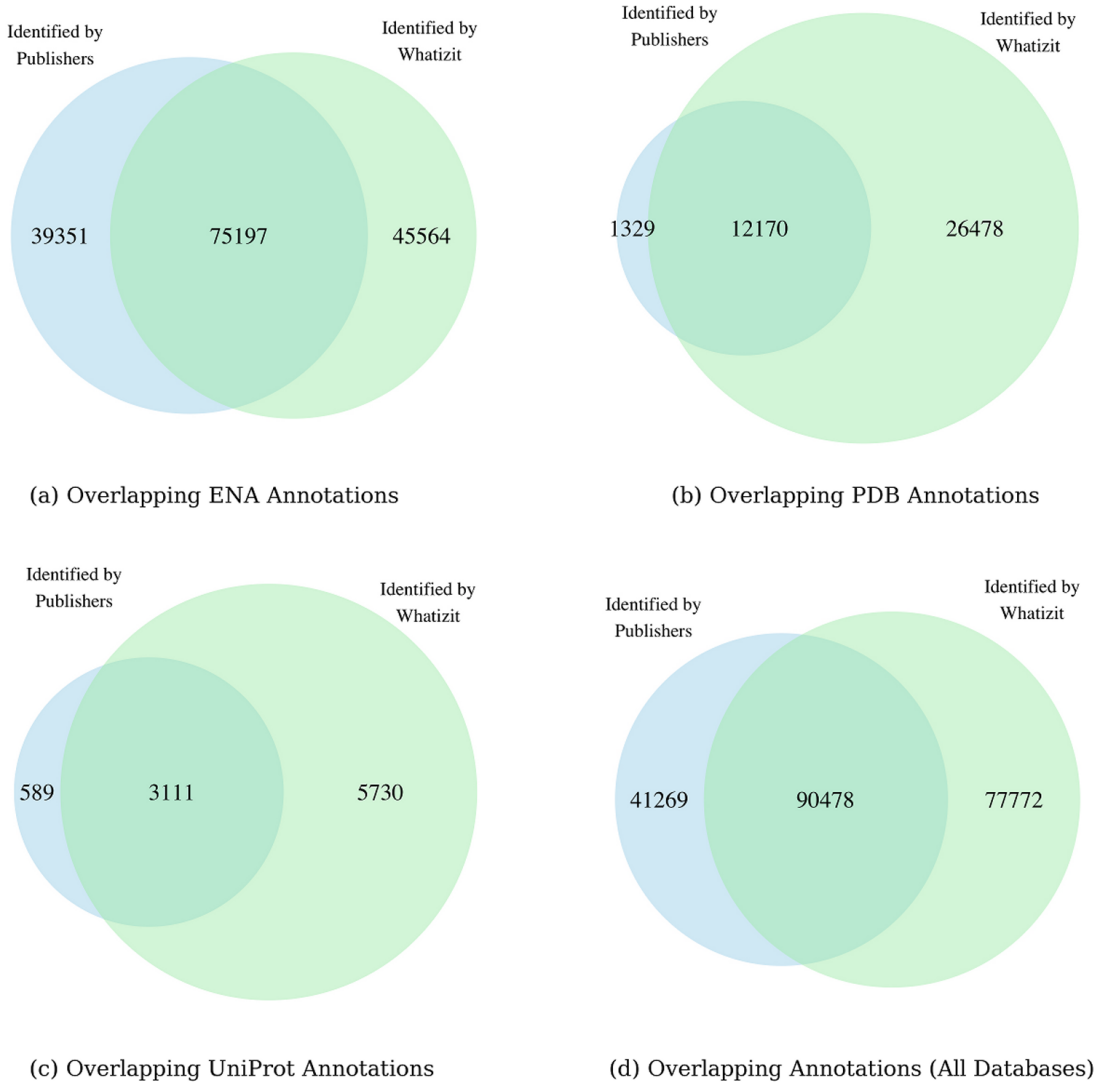
Venn diagrams showing the spread of accession numbers supplied in the article XML and annotated by the Whatizit ANA pipeline. (a) ENA (total: 160,112) (b) PDB (total: 39,972) (c) UniProt (total: 9,430) (d) all (total: 209,519). The results show that text mining substantially increases the number of accession numbers identified.

The majority of the accession numbers found in text are for ENA (Figure 4), likely reflecting the fact that nucleotide sequencing is a fundamental part of experimental biology, as well as how nucleotide database citation in research articles is embedded in the community. However, when the number of citations is expressed relative to the total number of entries in the source database, PDB accession numbers are cited at the highest rate, indicating significant reuse of PDB structures in the community (Table 3).

**Table 3:** Database citations in articles relative to database size.

Database	Total number of Annotations*	Total number of records in database (approx)	Citations per database record	Examples of most frequently cited accession numbers
ENA	160112	266 million	0.0006	AF009606 (Hepatitis C virus subtype 1a polyprotein gene, complete cds), AL513382 (Salmonella enterica subsp. enterica serovar Typhi str. CT18, complete chromosome)
PDB	39977	86000	0.46	1F66 (2.6 A Crystal structure of a nucleosome core particle containing the variant histone H2A.Z), 1LFD (Crystal structure of the active ras protein complexed with ras-interacting domain of ralgds)
UniProt	9430	540000**	0.017	P02768 (Serum albumin), P04406 (Glyceraldehyde-3-phosphate dehydrogenase GAPDH)
Total	209519	–	–	–

* Total number of Annotations =  Publisher-annotated + text-mined.

** This is the number of records in the curated component of UniProt.

When the results of publisher-tagged database citations are sorted according to journal title, it is clear that efficacy of mark-up is split mostly into two groups: those journal titles that do undertake mark-up of database citations and those that don't, with a few journal titles doing a partial annotation job. Those journal titles that do annotate, usually do it very well, identifying over 98% of possible accession numbers; those that don't generally annotate zero accession numbers, suggesting this is not a component of the journal mark-up system (see File S1).

In all three databases, the Whatizit ANA pipeline identified the majority of the accession numbers annotated by publishers (12,170 out of 13,499 for PDB (90%); 75,197 out of 114,548 for ENA (66%); 3,111 out of 3,700 for UniProt (84%)). The reasons for these false negatives from the Whatizit ANA pipeline are described in the methods section.

In addition, the Whatizit ANA pipeline annotated significant numbers of additional accession numbers for each database (26,478 for PDB (66% of total); 45,564 for ENA (47% of total); 5,730 for UniProt (61% of total)). This demonstrates that text mining significantly enriches the source XML with respect to identifying citations to these three databases.

### Accession number citations over time

**Figure 5:**
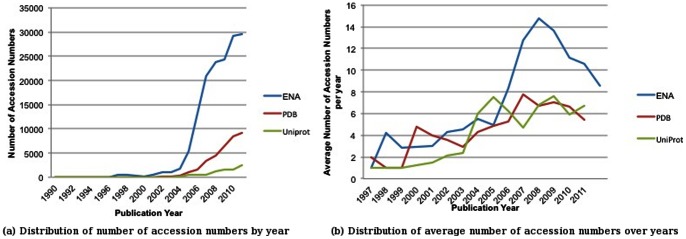
Distributions of number of and average number of accession numbers cited per article over time. The graphs show the number of (a) and the average number of (b) ENA, PDB and UniProt accession numbers cited per article according to publication year (in the OA-ePMC). Data from 2012 is excluded as it is not a complete year. In Figure 5(b), for a given year and database, average value is calculated by using articles containing accession number citations only. Text-mined results are used together with the publisher-annotated data to generate the graphs.

Figure 5 shows (a) the total and (b) the average numbers of accession numbers cited per article by publication year. As can be seen in Figure 5(a), the total number of accession numbers cited in articles per year increases as the number of articles increases. Figure 5(b) shows that a peak in the citation of nucleotide records around 2008, while citation of UniProt and PDB remain relatively constant or modestly increased in the same time period. The reason for the drop in the number of nucleotide citations is unclear. One explanation could be artefactual: the OA-ePMC dataset is a fraction of all articles published. Furthermore, if there is an increase in the number of articles citing small numbers of nucleotide accession numbers, then this may effect the averages shown in Figure 5(b). Whether these observations reflect any trends in the nature of core molecular biology research will require further investigation.

### Comparing accession numbers cited in the literature with article citation in databases

**Figure 6:**
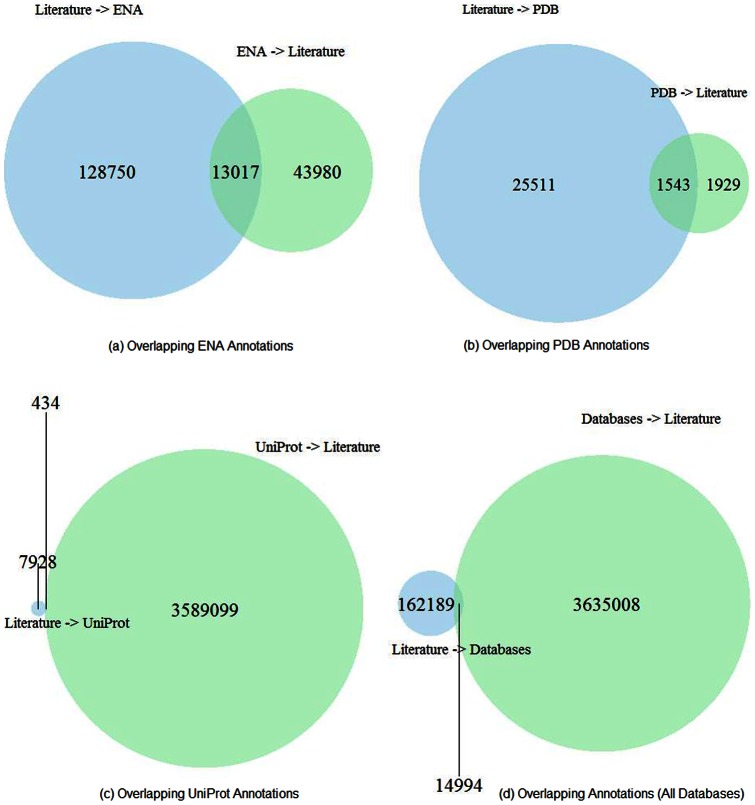
Comparison between article-to-database and database-to-citations. Venn diagrams show the overlapping article-to-database and database-to-article citations. (a) ENA (b) PDB (c) UniProt (d) all databases. Notable is that in the cases for ENA and PDB, database citations from the literature significantly enrich the database-literature crosslinks supplied from databases. For UniProt, the citations from the database to the literature dwarf the converse citations, mainly due to the fact that, for certain proteomes, many thousands of UniProt records can link to a single article. Text-mined results are used together with the publisher-annotated data to generate the venn diagrams.

It is common practice in biomolecular databases to cite the literature, either as part of the metadata for a database submission, or added by database curators as an ongoing activity. We compared these database-to-article links with the article-to-database references from the OA set of ePMC (Figure 6).

Only tiny portions of the database-to-article citations are in common with the article-to- database ones (14,994 of 3,650,002 (0.41%)). However, the results show that accession numbers gathered from the literature (article-to-database) could significantly help to improve literature-databases integration efforts.

A study carried out by Névéol and colleagues addressed the automatic extraction of links between the two biological databases (PDB and GEO) and the literature using both full text and Medline abstracts [6]. Similarly they have shown that text mining can help to improve the links between the biological databases and the literature. However, it is not possible to directly compare our study to their work since the total number of accession numbers used in their method is different from ours where both publisher annotated and text mined terms found from the literature are considered.

## Conclusion and Future Work

The OA subset of ePMC is now close to 500 K articles, representing a growing subset of articles indexed in PubMed. We used this subset to investigate data citation to three major databases (ENA, PDB and UniProt) in articles and in the process made several interesting findings:

- Data cited in articles is not always represented in structured form in the archival version.
- Text mining substantially extends the tagging of accession numbers, both in terms of number of those that often appear in structured form (UniProt, ENA and PDB) and newer databases (e.g. ArrayExpress, Ensembl, Pfam). The approach could be extended to other databases such as RefSeqs, or reference SNPs (rs numbers).
- The quality of the Whatizit ANA pipeline is high, with a focus on high precision (100%). The F-score values are above 95% for PDB and UniProt, and above 75% of F-score is obtained for ENA. The reasons for missed accession numbers are clear (see Section 2.4) and will be addressed in future releases of the pipeline.
- Patterns of citation vary between databases. For example, the citation pattern of PDBe accession numbers suggests high levels of data reuse.
- Text mining accession numbers with high precision makes a substantial contribution to the integration of the literature with related databases.
- There is an apparent drop in the citation of nucleotide database accession numbers since around 2008. The reasons for this require further investigation.

In the future, we will continue to improve and extend the Whatizit ANA pipeline and apply it to the full content of ePMC as part of the ongoing annotation effort. As this OA-ePMC set represents much less than 50% of content from the past 10 years, we expect the number of database citations gleaned in this way to grow significantly. These links will be made publically available via ePMC services.

On a broader scale, these findings suggest that as a community we are far from managing data citation in a manner that allows the development of tools and services such as data citation metrics, or other integrative applications. Text-mining can be used to extend structured data citation, and could be a basis for the development of services to help authors or editors to add structured content at the beginning of the publication process, rather than after the fact. Furthermore, feeding this citation information back to the source databases provides leads for database curators and contributes to the deeper integration of public data resources.

### Outcomes

- All the data described in this article are available at http://europepmc.org/ftp/oa/AccNoAnalysisData/.
- The Whatizit ANA pipeline for ENA, UniProt and PDB accession numbers is integrated into the ePMC infrastructure and all the gathered accession numbers are available via the ePMC web site and web services (http://europepmc.org/WebServices).
- The extensions and improvements to the Whatizit ANA pipeline will be applied to the ePMC core program of named entity recognition and will be available via the web site and web services.
- Tagged versions of the OA article set will be made available on an ongoing basis from the FTP site in the future.
- Accession numbers mined from articles will be fed back to the source databases to further improve the integration of literature with data.

## Supporting Information

File S1 **Journal based analysis of the OA-PMC articles.** This file presents distribution of the articles as well as the publisher-annotated articles based on the journals in the OA-PMC set.
(XLSX)
